# Autochthonous West Nile virus infection outbreak in humans, Leipzig, Germany, August to September 2020

**DOI:** 10.2807/1560-7917.ES.2020.25.46.2001786

**Published:** 2020-11-19

**Authors:** Corinna Pietsch, Dominik Michalski, Johannes Münch, Sirak Petros, Sandra Bergs, Henning Trawinski, Christoph Lübbert, Uwe G Liebert

**Affiliations:** 1 Institute of Virology, Leipzig University Hospital, Leipzig, Germany; Interdisciplinary Centre for Infectious Diseases, Leipzig University Hospital, Leipzig, Germany; 2 Department of Neurology, Leipzig University Hospital, Leipzig, Germany; 3 Division of Nephrology, Department of Endocrinology, Nephrology and Rheumatology, Leipzig University Hospital, Leipzig, Germany; 4 Medical ICU, Leipzig University Hospital, Leipzig, Germany; 5 Division of Infectious Diseases and Tropical Medicine, Department of Medicine II, Leipzig University Hospital, Leipzig, Germany; Interdisciplinary Centre for Infectious Diseases, Leipzig University Hospital, Leipzig, Germany

**Keywords:** Viral encephalitis, zoonoses, arboviruses, Outbreak, West Nile neuroinvasive disease, West Nile fever

## Abstract

Following a distinct summer heat wave, nine autochthonous cases of West Nile fever and West Nile neuroinvasive disease, including one fatality, were observed in Leipzig, Germany, in August and September 2020. Phylogenetic analysis demonstrated close relationships in viruses from humans, animals and mosquitos in eastern Germany, obtained during the preceding 2 years. The described large cluster of autochthonous West Nile virus infections in Germany indicates endemic seasonal circulation of lineage 2 viruses in the area.

In August and September 2020, seven cases of West Nile neuroinvasive disease (WNND) and two cases of West Nile fever (WNF) were identified in patients at the Leipzig University Hospital, in eastern Germany. Five cases fulfilled the European Union case definitions for laboratory confirmed WNV infections and four cases were classified as probable WNV infections [1]. Here we describe the cases with their clinical characteristics and exact virological findings.

## Cases’ characteristics

Symptom onset in the seven male and two female cases occurred between 15 August and 9 September. All cases had geographic links to the city of Leipzig or to the surrounding district of Leipzig. None of them travelled abroad during the 4 weeks before symptom onset (Table 1). Six of the WNND cases were older than 60 years and three received immunosuppressive treatment. The WNND cases showed clinical, radiological, and laboratory features of meningitis, meningoencephalitis, encephalitis or acute flaccid paralysis. Additionally, fever, diarrhoea, rash, lymphadenopathy, renal failure, hepatitis and sepsis were observed. One case died (Table 1).

**Table 1:** Characteristics of human West Nile virus infection cases^a^, Leipzig, Germany, August–September 2020 (n = 9)

Cases	Case classification	Disease	Symptom onset (2020)	Age (years)	Recent travel	Immunosuppressive medication	Clinical manifestations
**Case 1**	Probable	WNND	Aug	30–49	None	Yes^b^	Fever, rash, lymphadenopathy, headache, neck pain, phono- and photophobia
**Case 2**	Confirmed	WNND	Aug	80–89	Federal State of Brandenburg, North-eastern Germany	No	Fever, sepsis, renal failure, impaired consciousness
**Case 3**	Confirmed	WNND	Aug	60–69	None	Yes^b^	Fever, diarrhoea, headache, impaired consciousness
**Case 4**	Confirmed	WNND	Aug	70–79	None	No	Fever, impaired consciousness, seizures
**Case 5**	Confirmed	WNND	Aug	70–79	None	Yes^b^	Fever, diarrhoea, vomiting, orientation disorder, renal failure, impaired consciousness
**Case 6**	Confirmed	WNND	Aug	70–79	Federal State of Saxony-Anhalt, Central Germany	No	Fever, diarrhoea, renal failure, hepatitis, tetraplegia, persistent coma, death
**Case 7**	Probable	WNND	Sep	60–70	None	No	Acute flaccid paralysis of the lower extremities
**Case 8**	Probable	WNF	Sep	30–39	Leipzig District, Western Saxony	No	Rash, nausea, diarrhoea, head and body aches, photophobia
**Case 9**	Probable	WNF	Sep	10–20	Leipzig District, Western Saxony	No	Rash, nausea, diarrhoea, head and body aches, photophobia

WNF: West Nile fever; WNND: West Nile neuroinvasive disease.

^a^ Five confirmed and four probable cases according to the European Union case definition [1].

^b^ Azathioprine or prednisolone.

## Laboratory findings and phylogenetic analysis of genetic sequences

A summary of laboratory findings is provided in Table 2. All cases showed positive results in WNV IgM ELISA (Euroimmun, Lübeck, Germany). Except for one of the cases receiving immunosuppressive medication, WNV IgG (Euroimmun) was either present in initial serum samples or seroconversion was documented by analysis of follow-up samples. Clinical samples from five cases revealed WNV RNA by real-time reverse-transcription PCR (RT-PCR) [2]. Of these, WNV RNA was found in urine in five cases, in serum in four and in cerebrospinal fluid (CSF) in one case. To demonstrate presence of infectious virus particles, WNV was isolated on Vero cells from three urine samples with high viral load [3].

**Table 2:** Virological data of human West Nile virus infection cases^a^, Leipzig, Germany, August–September 2020 (n = 9)

Cases	Time to diagnosis (days)^b^	WNV IgM	WNV IgG	WNV RNA in urine (GE/mL)	WNV RNA in serum (GE/mL)	WNV RNA in CSF (GE/mL)	WNV cell-culture isolation	WNV lineage
**Case 1**	8	Positive	Sero-conversion	Undetectable	Undetectable	Undetectable	ND	ND
**Case 2**	6	Positive	Positive	1,010,000	1,000	Undetectable	Positive	2
**Case 3**	6	Positive	Negative^c^	500	200	Undetectable	ND	2
**Case 4**	6	Positive	Sero-conversion	100	Undetectable	Undetectable	ND	2
**Case 5**	6	Positive	Positive	6,450,000	150	Undetectable	Positive	2
**Case 6**	7	Positive	Sero-conversion	67,200,000	5,200	1,500	Positive	2
**Case 7**	8	Positive	Positive	Undetectable	Undetectable	Undetectable	ND	ND
**Case 8**	5	Positive	Sero-conversion	Undetectable	Undetectable	ND	ND	ND
**Case 9**	12	Positive	Positive	Undetectable	Undetectable	ND	ND	ND

CSF: cerebrospinal fluid; GE: genome equivalents; ND: not determined; WNV: West Nile virus.

^a^ Five confirmed and four probable cases according to the European Union case definition [1].

^b^ This is the time between symptom onset and the day of first sample collection.

^c^ WNV IgG was still undetectable at the last day of follow-up (20 days after symptom onset).

The WNV-IgM positive sera were also tested for tick-borne encephalitis virus (TBEV) IgM antibodies (Euroimmun, Lübeck, Germany). The TBEV-IgM ELISA was non-reactive in all of them. TBEV-IgG was either negative (n = 3), high positive (n = 3) or low positive (n = 3). Thereof, all patients with history of TBE vaccination showed high TBEV-IgG titres. As no significant increase of the antibody titre was observed in follow-up samples of the low positive TBEV-IgG cases, the latter results were interpreted as probable cross-reactivity of the assay with the present WNV-IgG or with any other pre-existing flavivirus antibodies, e.g. against Japanese encephalitis, Dengue or Yellow fever. All available CSF samples from the cases were negative for TBEV-RNA by real-time RT-PCR.

**Figure 1:**
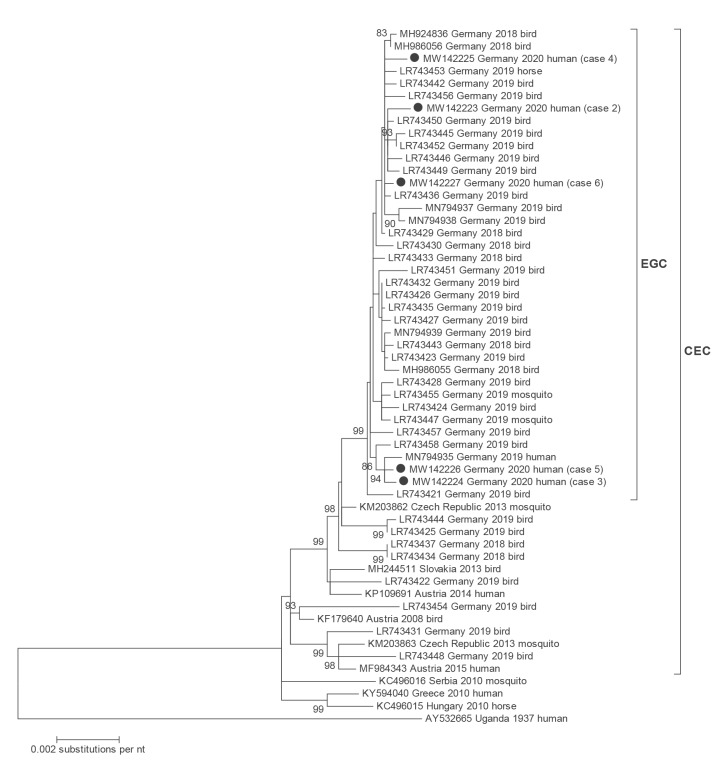
Phylogenetic analysis of almost complete WNV lineage 2 genomes (11,005 nt)

CEC: Central and Eastern European clade; EGC: Eastern German clade; nt: nucleotides; WNV: West Nile virus.

Full-coding viral genomes were assessed using Sanger sequencing and primer walking for four of the WNV cases with detectable viral RNA. Due to the very low viral load, only partial genome sequences were derived from the fifth one (GenBank accession numbers MW142223 to MW142227). Sequences were aligned in Geneious Prime software (Biomatters, Auckland, New Zealand). Phylogenetic analysis was conducted using MEGA 5 [4]. All obtained genome sequences showed lineage 2 viruses. Phylogenetic analysis demonstrated their close genetic relationship to viral strains detected in humans, birds and horses in eastern Germany during the 2 preceding years (Figure 1).

All WNV strains sequenced in this study are indicated by filled circles and are part of the main CEC – which includes the EGC as proposed earlier by Ziegler et al. [17]. Nucleotide identity between the present WNV lineage 2 genomes was 99.8–99.9%. The evolutionary history was inferred by using the Maximum Likelihood method based on the Tamura–Nei model. GenBank accession numbers of included sequences are shown on the tree. The scale bar indicates nt substitutions per site. Bootstraps values above 80% are shown next to the branches.

## Meteorological parameters and environmental situation in the city of Leipzig

**Figure 2:**
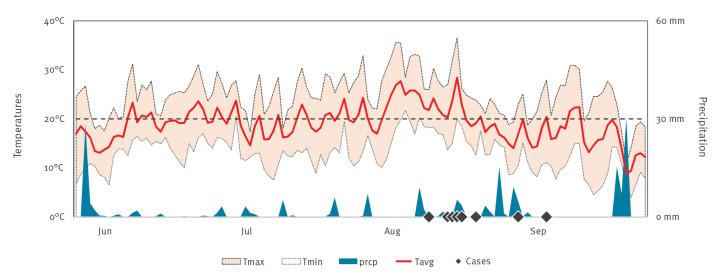
Temperatures and precipitation in the city of Leipzig, June–September 2020

Leipzig is a city of 600,000 inhabitants in central-eastern Germany and belongs to the Federal State of Saxony. Overall, Leipzig has a temperate seasonal climate with relatively mild winters and warm rather than hot summers. The warmest month, on average, is July with an average temperature of 18°C (Leipzig Institute for Meteorology, Faculty of Physics and Earth Sciences, Leipzig University). In 2020, there was a heat wave with average daily temperatures above 20 °C from 6 August to 22 August. In parallel, precipitation was below average (ca 60 mm) with a monthly accumulated rainfall of 22.2 mm and 50.1 mm in July and August, respectively (Figure 2).

Daily average temperatures (Tavg), maximum temperatures (Tmax), minimum temperatures (Tmin) and precipitation (prcp) from June to September 2020 are shown. Source: meteostat.net. Black diamonds indicate the days of symptom onset of the West Nile virus infection cases.

Irrespectively of the dry period in summer 2020, the Leipzig urban area provides multiple natural freshwater habitats for mosquitoes such as wetlands, large alluvial forests, park ponds, confluent rivers, large pit lakes, and numerous allotment gardens.

### Ethical statement

The Leipzig University Ethics Committee approved the study (no. AZ 298/16‐ek). Only anonymous patient and ward data are presented to protect the patients’ identity.

## Discussion and conclusions

West Nile virus is maintained in nature through an enzootic bird–mosquito cycle. Transmitted by different species of *Culex* mosquitoes, it can affect a wide range of accidental hosts such as horses and humans. Different lineages are circulating [5]. In 2004, WNV lineage 2 emerged in Europe [6]. Today, WNV lineage 2 is endemic in many European countries and causes seasonal regional outbreaks with increasing frequency [7,8].

Imported WNV infections were observed earlier in Germany [9,10], but autochthonous transmission of WNV in the country was diagnosed as late as in 2018 for the first time, in resident birds and horses in central-eastern Germany [11]. Prior to that, the virus had been detected neither in potential reservoir species nor in vectors in spite of a comprehensive nationwide wild bird surveillance system [12,13] and an exhaustive mosquito surveillance programme for zoonotic arthropod-borne viruses [14-16]. The number of WNV-positive birds and horses rose considerably in 2019 and five autochthonous human WNV infection cases were observed [17]. At the same time, WNV was demonstrated in indigenous *Culex* spp [18]. The areas with highest activity of WNV were the German Federal States of Saxony, Saxony-Anhalt, Brandenburg and Berlin, i.e. central-eastern Germany [17]. Notably, all autochthonous human cases occurred in this region – three of them in the municipal area of Leipzig [17]. All genetically characterised viruses detected from human and animal cases and vectors in 2018 and 2019, showed close phylogenetic links to the Eastern European clade of WNV lineage 2 viruses [17].

In contrast to the five sporadic human WNV cases detected in 2019, the presented cluster of nine autochthonous WNND and WNF cases was detected at a single hospital within an observation period of less than 4 weeks. Based on the cases’ place of residence and travel history, the presence of an endemic cycle in local species of birds and mosquitos in the city of Leipzig has to be assumed. This assumption is also supported by the common spatial link between human WNV cases in 2020 and 2019. Furthermore, phylogenetic analysis demonstrated that WNV in central-eastern Germany has maintained genetically stable since the first detection in 2018. This makes new WNV introductions via migratory birds an unlikely scenario and rather indicates WNV lineage 2 persistence in resident reservoir bird species or hibernating mosquitoes.

The present cases occurred in late summer after a distinct hot and dry period. It is known that high daily average temperatures (> 20 °C) over several days are required to allow for efficient WNV transmission [19]. Thus, the 2020 summer heat wave presumably drove the epizootic emergence of the virus in Leipzig by shortening the extrinsic incubation period of the virus in the vector mosquitoes. In addition, increased WNV activity is rather related to dry conditions than to rainy periods [20]. Among others, a possible reason for this is the congregation of reservoir host birds around dwindling water sources, which increases the rates of contact between avian reservoirs and mosquitoes and thereby virus transmission [20].

There was a wide age-range among our cases. Advanced age remains a major risk factor for disease caused by WNV and individuals older than 50 years of age are more susceptible to severe infections with neurological involvement [5]. Accordingly, the majority of the WNND cases documented here occurred among people aged 60 years and older. Given that less than 1% of infected humans develop WNND [5], it seems reasonable to assume that the local outbreak of WNV infections may have affected several hundred people. Presumably, most of them developed only minor or no clinical symptoms at all. This assumption is well supported by the fact that WNV infections have recently been identified in asymptomatic blood donors in the area [21].

In conclusion, the presented WNV infections cluster represents so far the largest outbreak of autochthonous human cases in Germany, to the best of our knowledge. An endemic seasonal circulation of WNV lineage 2 viruses in the city of Leipzig and the surrounding districts has to be assumed. During the coming summers, epizootic re-emergence of the virus and increasing case numbers in humans are possible. Therefore, awareness of human WNV infections should be further increased in the general population and among healthcare workers i.e. neurologists, internists and general practitioners, by education programmes. Moreover, in the authors’ opinion, existing surveillance programmes for birds and mosquitoes should be expanded and intensified in Leipzig and the surrounding districts.
